# A Fluorescence-Based Method for Rapid and Direct Determination of Polybrominated Diphenyl Ethers in Water

**DOI:** 10.1155/2015/853085

**Published:** 2015-01-29

**Authors:** Huimei Shan, Chongxuan Liu, Zheming Wang, Teng Ma, Jianying Shang, Duoqiang Pan

**Affiliations:** ^1^Laboratory of Basin and Wetland Eco-Restoration, China University of Geosciences, Wuhan 430074, China; ^2^Pacific Northwest National Laboratory, Richland, WA 99352, USA; ^3^State Key Laboratory of Biogeology and Environmental Geology, China University of Geosciences, Wuhan 430074, China

## Abstract

A new method was developed for rapid and direct measurement of polybrominated diphenyl ethers (PBDEs) in aqueous samples using fluorescence spectroscopy. The fluorescence spectra of tri- to deca-BDE (BDE 28, 47, 99, 153, 190, and 209) commonly found in environment were measured at variable emission and excitation wavelengths. The results revealed that the PBDEs have distinct fluorescence spectral profiles and peak positions that can be exploited to identify these species and determine their concentrations in aqueous solutions. The detection limits as determined in deionized water spiked with PBDEs are 1.71–5.82 ng/L for BDE 28, BDE 47, BDE 190, and BDE 209 and 45.55–69.95 ng/L for BDE 99 and BDE 153. The effects of environmental variables including pH, humic substance, and groundwater chemical composition on PBDEs measurements were also investigated. These environmental variables affected fluorescence intensity, but their effect can be corrected through linear additivity and separation of spectral signal contribution. Compared with conventional GC-based analytical methods, the fluorescence spectroscopy method is more efficient as it only uses a small amount of samples (2–4 mL), avoids lengthy complicated concentration and extraction steps, and has a low detection limit of a few ng/L.

## 1. Introduction

Polybrominated diphenyl ethers (PBDEs) are emerging surface and groundwater contaminants that have received increasing public and regulatory scrutiny and thus research interest [1]. PBDEs are a class of organobromine compounds widely used as flame retardants (BFRs) that are commonly mixed into various industrial and household products including building materials, electronics, plastics, and foams. The BFRs become contaminants as they dissociate from such products and are released into environment [1]. Although PBDEs have been phased out in Europe and voluntarily withdrawn from the market in the US, they are still widely detected in soil, sediment, surface waters, air, animal, and human body [2–9]. PBDEs are lipophilic and hydrophobic with a strong affinity to solid materials. Despite this property, they are still detected in both surface water [10, 11] and groundwater [12]. Increasing evidences indicate that PBDEs are endocrine disruptors possessing toxicity to liver, thyroid, and neurodevelopment [9, 13–15].

The concentrations of PBDEs in aqueous samples are commonly determined using gas chromatography (GC) approaches with electron capture detector (ECD) [16], GC coupled to mass spectrometry (MS) with either negative chemical ionization (NCI) or electron impact (EI) as ionization techniques [17–19]. All these methods, however, require pretreatment of aqueous samples through solvent extraction, concentration, and purification. In addition, GC-based approach may be compromised when high-Br PBDEs congeners (e.g., BDE 209) degrade to low-Br PBDEs congeners at high temperature in chromatography column.

Here we report a new method to analyze PBDEs in water samples that can avoid the shortcomings in the GC-based approaches. The new method is based on characteristic fluorescence properties of PBDEs under room temperature. Although fluorescence-based methods have been widely used in analyzing hydrophobic organic compounds in aqueous phase [20–23], sediments [24, 25], and engineered nanoporous materials [26], its application to analyze PBDEs has not been reported to our knowledge. The major objectives of this study are therefore to (1) characterize fluorescent properties of six major PBDEs congeners that are commonly found in environment and (2) demonstrate the efficacy of the fluorescence spectroscopy method for measuring the concentrations of PBDEs in aqueous samples. The new method was also compared with the GC-based approaches (GC-ECD and GC-EI-MS) to identify the advantages and disadvantages of the approaches.

## 2. Materials and Methods

### 2.1. Chemicals

**Figure 1:**
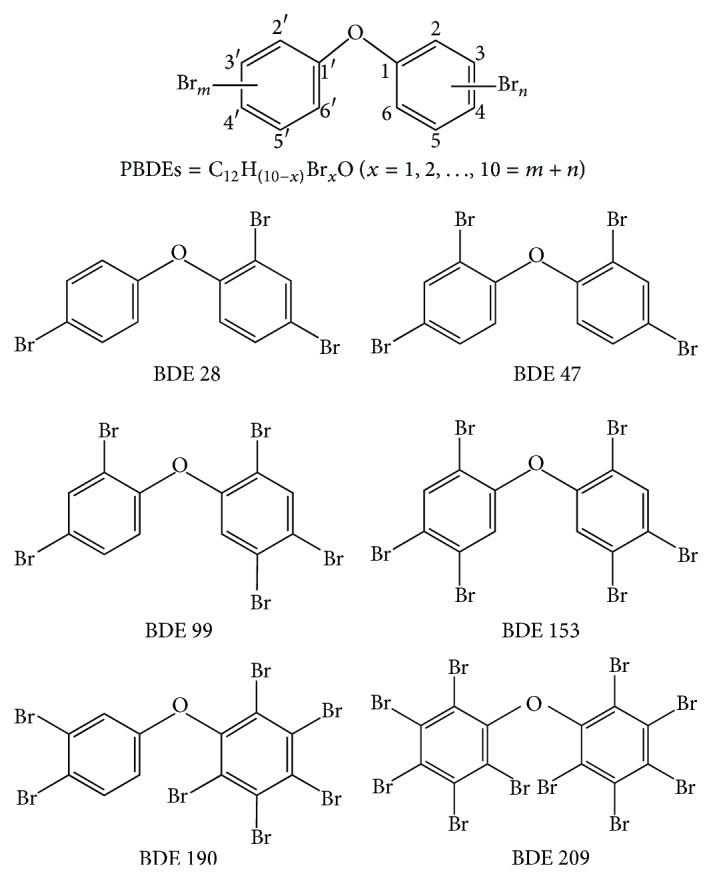
The chemical structures of six major PBDEs including BDE 28, BDE 47, BDE 99, BDE 153, BDE 190, and BDE 209.

Six congeners of PBDEs including 2,4,4′-tribromodiphenyl ether (BDE 28), 2,2′,4,4′-tetrabromodiphenyl ether (BDE 47), 2,2′,4,4′,5-pentabromodiphenyl ether (BDE 99), 2,2′,4,4′5,5′-hexabromodiphenyl ether (BDE 153), 2,3,3′,4,4′,5,6-heptabromodiphenyl ether (BDE 190), and decabromodiphenyl ether (BDE 209) were purchased from AccuStandard (New Haven, CT, USA). Their chemical structures are provided in Figure 1. The stock solutions of these chemicals (1 × 10^6^ ng/L) were prepared by mixing 1 mL PBDEs (5 × 10^7^ ng/L in isooctane) and 49 mL ethanol solution (≥99.5%, Fisher Scientific). The high mass ratio of ethanol used in the stock solution is to guarantee that the PBDEs and isooctane will completely mix with water in preparing standard solutions as described below. The stock solutions were diluted using deionized water (DI water) to prepare work solutions of 1000 ng/L for tri- to hexa-BDE and 100 ng/L for hepta- and deca-BDE, which were then used to prepare series of standard solutions of 1000, 200, 40, 8, 1.6, 0.32, and 0.064 ng/L for tri- to hexa-BDE and 100, 50, 25, 5, 1, 0.2, and 0.04 ng/L for hepta- and deca-BDE in DI water. Humic acid was purchased from Fisher Scientific (≥90%, MP Biomedicals) and used as received. 0.2 g/L HA solution was obtained by dissolving 0.2 g humic acid in 1 L DI water by constant stirring for 48 h.

### 2.2. Fluorescence Measurements

The fluorescence spectra of PBDEs in aqueous samples were recorded using a conventional fluorimeter (Fluorolog III, Horiba Jobin Yvon Inc., Edison, NJ) equipped with a 350 W xenon lamp in quartz cuvettes (3 mL) and a Hamamatsu R928 photomultiplier tube at −950 V. The excitation wavelength (*λ*_exc_, nm) was varied from 240 to 360 nm and emission wavelength (*λ*_em_, nm) was from 350 to 580 nm in intervals of 1 nm and exposure time of 0.1 second. Linear correlation of the intensity or integral areas of the characteristic fluorescence peaks with corresponding PBDEs concentrations was then established for each PBDEs congener and the detection limit was determined for the method.

### 2.3. Effects of Environmental Variables on Fluorescence Measurement

The effects of pH and humic substance on the fluorescence method were evaluated using BDE 47. BDE 47 was selected because it is the predominant congener of PBDEs in surface and groundwaters with a concentration ranging from pg/L to tens of ng/L [2, 10, 11, 27, 28]. The pH in the PBDEs solutions was adjusted by acid-base titration using 1 mM HCl or 1 mM NaOH. The effect of humic substance on fluorescence measurements was evaluated by successively adding aliquots of 200 mg/L humic acid in BDE 47 solutions.

### 2.4. Effect of Groundwater Chemical Composition

Synthetic groundwater (SGW) with the same chemical composition as that in groundwater at the U.S. Department of Energy's Hanford site, Washington State (pH 8.1 and ionic strength 6.3 mM) [29], was used as a background solution instead of DI water to evaluate the potential interference of inorganic groundwater chemical constituents on PBDEs fluorescence measurements. BDE 47 stock solution (1 × 10^6^ ng/L) was mixed into the SGW to prepare a series of solutions ranging from 64 to 200 ng/L of BDE 47. The resulting solutions were stirred for more than 10 h with the sample containers wrapped with aluminum foil to avoid light exposure. After mixing, the solutions were measured to establish the relationship between fluorescence intensity and BDE 47 concentration in SGW. The relationship was compared to that derived in DI water to evaluate the effect of background inorganic solution chemistry on the PBDEs measurements.

## 3. Results and Discussion

### 3.1. Fluorescence Characteristics of PBDEs

**Figure 2:**
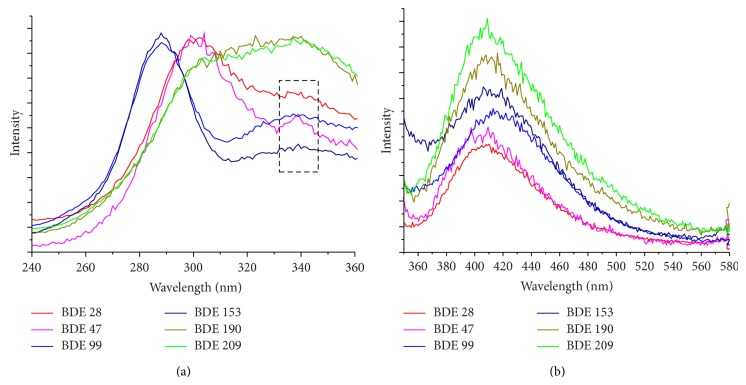
Excitationspectra (a) and emission spectra (b) of BDE 28, BDE 47, BDE 99, BDE 153, BDE 190, and BDE 209. The excitation spectra were collected at the emission wavelength (*λ*_em_) of 440 nm and the emission fluorescence spectra were collected at excitation wavelength (*λ*_exc_) of 302 nm.

The excitation spectra of the PBDEs recorded at fluorescence emission wavelength (*λ*_em_) of 440 nm (Figure 2(a)) show behavior of three groups: 2,4,4′-tribromodiphenyl ether (BDE 28) and 2,2′,4,4′-tetrabromodiphenyl ether (BDE 47) formed the first group, which displays a major peak near *λ*_exc_ = 302 nm; 2,2′,4,4′,5-pentabromodiphenyl ether (BDE 99) and 2,2′,4,4′,5,5′-hexabromodiphenyl ether (BDE 153) formed the second group with a major peak near *λ*_exc_ = 289 nm; 2,3,3′,4,4′,5,6-heptabromodiphenyl ether (BDE 190) and decabromodiphenyl ether (BDE 209) formed the third group with a plateau from *λ*_exc_ = 320 to 340 nm. A minor peak near *λ*_exc_ = 335 was also observed for PBDEs with small number of bromine (BDE 28, BDE 47, BDE 99, and BDE 153) (Figure 2(a)). The behavior of the fluorescence excitation spectra of the three groups was also observed at other emission wavelengths (data not shown). These results indicated that the number and position of Br on the benzene rings of 3, 3′, 5, 6 (Figure 1) affected the excitation features of PBDEs apparently through influencing the original conjugated *π* or p-*π* system [30]. On the other hand, the result that PBDEs congeners within each group have similar spectra indicating that Br substituent position of 2′, 5′ and 6′ on the benzene rings (Figure 1) apparently did not have the effect on the excitation spectrum. The major peak position in each excitation spectrum was provided in Table 1 for different PBDEs at different emission wavelengths.

The emission spectra of PBDEs at variable excitation wavelengths (*λ*_exc_ = 289, 302, 320, and 340 nm) were also collected, and Figure 2(b) shows an example of the emission spectra collected at *λ*_exc_ = 302 nm. Emission spectra collected at other excitation wavelengths were similar (data not shown). Figure 2(b) shows that six PBDEs have similar emission fluorescence spectra, displaying a large peak near *λ*_em_ = 407 to 420 nm. The emission peak positions generally shifted right with increasing excitation wavelength (Table 1). Minor difference in emission peak position, however, exists for different PBDEs and the difference in their peak positions changes with excitation wavelength (Table 1). These properties, in combination with excitation spectra, can be used to distinguish different congeners in aqueous samples.

### 3.2. Linearity, Reproducibility, and Repeatability

The peak intensity and area in the fluorescence emission spectra were used to establish their relationships with PBDEs concentrations. In the data analysis, the fluorescence intensity or spectral area (*I*_*F*_ or ∑*I*_*F*_) was calculated as follows:

(1) $$\begin{array}{c} I_{F}=I_{Fi}-I_{Fo} \end{array}$$

or

(2) $$\begin{array}{c} \sum I_{F}=\sum I_{Fi}-\sum I_{Fo}, \end{array}$$

where *I*_*Fi*_ is the fluorescence peak intensity and *I*_*Fo*_ is the fluorescence intensity for background solvent. ∑*I*_*Fi*_ is the area surrounding a characteristic fluorescence peak, and ∑*I*_*Fo*_ is the area for background solvent.

**Figure 3:**
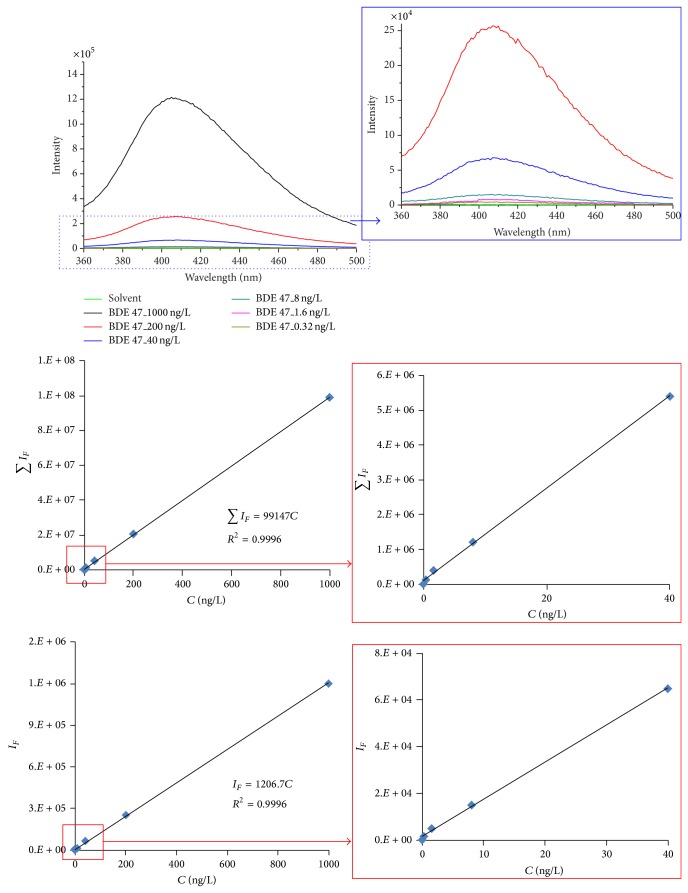
Emission spectra (*λ*_exc_ = 302 nm) as a function of BDE 47 concentration (0.32 ng/L to 1000 ng/L). The figure inserts show the linear correlation between the peak intensity *I*_*F*_ (at 406 nm) or peak area ∑*I*_*F*_ (from 360 to 500 nm) with BDE 47 concentration.

Figure 3 shows an example of the correlation between emission peak intensity or area and corresponding concentration of PBDEs measured at *λ*_exc_ = 302 nm using BDE 47 as the example. The strength or the area of the emission spectra peak increased with increasing PBDEs concentration. A good linearity was observed using either the spectral areas (*R* ^2^ = 0.9996) or the spectral peak intensity (*R* ^2^ = 0.9996). The linearity was observed for all other studied PBDEs congeners (BDE 28, BDE 99, BDE 153, BDE 190, and BDE 209) (spectra not shown). In addition, replicate sample analyses indicated that the method has a good reproducibility with mean relative standard deviation values lower than 4.74% in all cases.

### 3.3. Limit of Detection (LOD)

The detection limits were calculated as three times the standard deviation of ten replicate samples at concentration near the detection limit [31]. The calculated detection limits of BDE 28, BDE 47, BDE 99, BDE 153, BDE 190, and BDE 209 were 5.82, 2.70, 69.95, 45.55, 1.71, and 3.81 ng/L, respectively.

### 3.4. Effect of pH on Fluorescence Measurement

**Figure 4:**
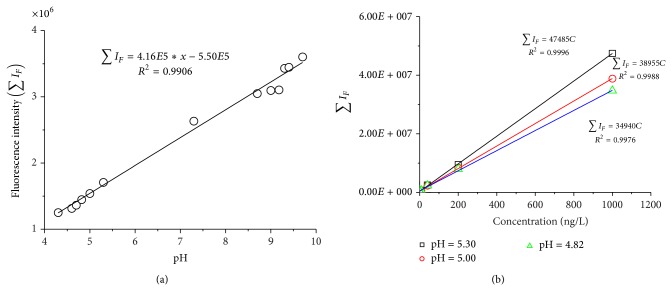
Fluorescence intensity (∑*I*_*F* 360~500 nm_) variations of 50 ng/L BDE 47 as a function of pH (a) and the influence of pH on the slope of the linear correlations between the fluorescence intensity and BDE 47 concentration (b).

Solution pH may affect fluorescence intensity of organic compounds [32–34].  Figure 4(a) shows that the fluorescence intensity of BDE 47 (50 ng/L in DI water) increased linearly with increasing pH, indicating that pH affects PBDEs measurements. The effect of pH on fluorescence intensity of organic compounds is typically attributed to the ionization effect [35] and the subsequent alternation of molecular structures of fluorophores. The effect of ionization is known to disappear near and above pH neutrality [36]. The consistent increase in fluorescence intensity with increasing pH (below and above the neutrality) (Figure 4(a)) suggested that the pH effect on the PBDE fluorescence properties was most likely caused by the changes in molecular configuration. Ghosh and Schnitzer [37] found that organic matter (e.g., humic substances) has a linear structure at high pH and forms coils when pH decreases. The coil structure may mask some internal fluorophores, leading to lower fluorescence emission at lower pH. At a higher pH, the configuration becomes linear and masked fluorophores are exposed to fluoresce, which increases the fluorescence intensity. The coil structure may be induced by strong H-bond between H^+^ and the fluorophores within the HA molecule. Such effect would be more pronounced at lower pH, where abundant H^+^ is available to form the H-bond. As pH increased (and [H^+^] drops), fewer H-bonds were formed and the deexcitation effect becomes weaker, leading to increased fluorescence intensity. The pH effect on the fluorescence intensity of PBDEs was fully reversible with increasing and decreasing pH cycles.

While pH affects PBDEs fluorescence intensity, the linear correlation between the fluorescence intensity and its concentration was held at all PBDE concentrations examined (Figure 4(b)). In other words, solution pH only affected the slope of the linear correlation curves between the fluorescence intensity and PBDEs concentration; the linear calibration curves are all valid at different pH, while the slope of the liner curves could be corrected at any given solution pH. These results indicated that solution pH is an important factor to consider in establishing calibration curve and/or adjusting pH in PBDEs samples in applying fluorescence method.

### 3.5. Effect of Humic Acid on Fluorescence Measurement

**Figure 5:**
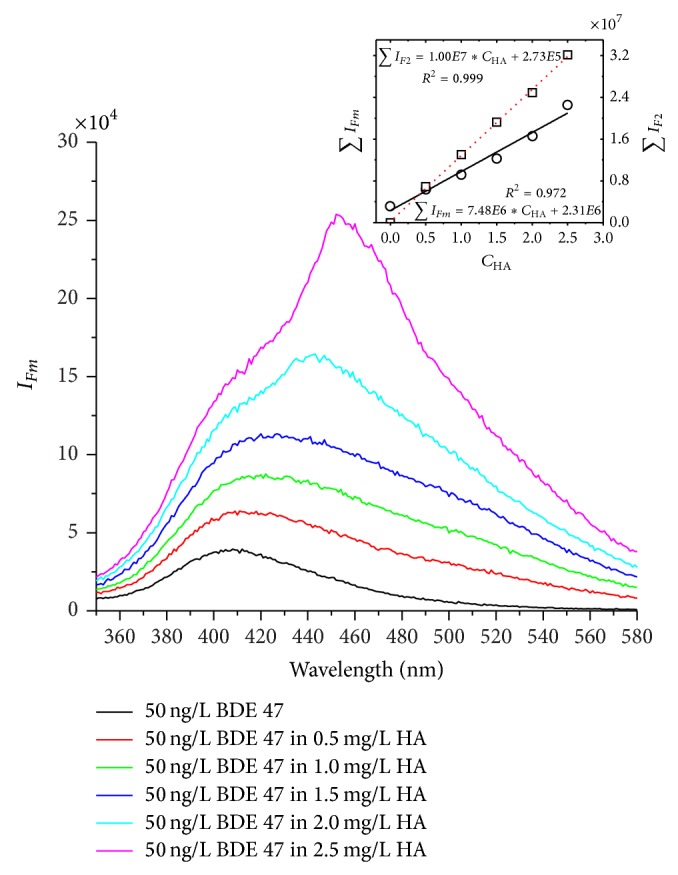
Fluorescence spectra of solutions containing mixed BDE 47 and HA. The figure inserts show the linear correlations of fluorescence intensity as a function HA concentration in solutions with and without BDE 47.

Humic substances, which contain fluorescent functional groups, are expected to interfere with the fluorescent determination of organic contaminants in aqueous samples [21, 23, 38–40].  Figure 5 shows that when the concentration of humic acid (HA) is 10^4^ time that of BDE 47, the measured overall fluorescence intensity at the emission maximum of BDE 47 (~408 nm) increased from 40,000 (a.u) to ~62,000 (a.u); that is, the net contribution from HA emission intensity was about half of that of BDE 47 (Figure 5). Therefore, any interference of fluorescence measurement of BDE 47 from HA will likely be insignificant or can be properly corrected at HA concentration levels <10^4^ times that of BDE 47. However, due to the partial overlap of the emission spectra of BDE 47 and that of humic acid (HA), the overall emission intensity of the BDE 47 and HA mixtures increased and the spectra became broader when HA concentration was further increased in solution (Figure 5). At such HA concentrations, the coexistence of HA and PBDEs significantly affected the fluorescence strength as revealed by lower fluorescence intensity in the mixed HA and PBDEs solutions than that in the solutions containing HA only when HA concentration was above 0.5 mg/L (Figure 5 insert). The linear relationship, however, still exists showing the overall fluorescence intensity increased with increasing HA concentration, suggesting that the fluorescence signal contribution from HA and PBDEs can be separated.

Assume that the measured fluorescence intensity of PBDEs solution containing HA is the sum of those from both PBDEs and HA, as follows,

(3) $$\begin{array}{c} \sum I_{Fm}=a*\sum I_{F1}+b*\sum I_{F2}, \end{array}$$

where ∑*I*_*Fm*_ is the measured peak area jointly contributed from PBDEs and HA, ∑*I*_*F*1_ is the fluorescence peak area contributed from PBDEs only and ∑*I*_*F*2_ is contributed from HA only, and *a* and *b* are the fitting parameters. By fitting the measured spectral data (Figure 5), *a* and *b* were determined to be 0.96 and 0.08 for BDE 47 solutions, respectively. A large *a* value and a small *b* value suggest that PBDEs had a stronger effect on HA fluorescence signal than the HA on PBDEs fluorescence. Considering that HA concentrations in environmental waters for a given area during a defined period of time in a year are relatively stable and can be properly measured based on total organic carbon concentrations, it is possible that the effect of HA on PBDEs quantification at HA concentration ≥10^4^ times that of PBDEs can be properly estimated and corrected.

### 3.6. Effect of Groundwater Chemical Composition

**Figure 6:**
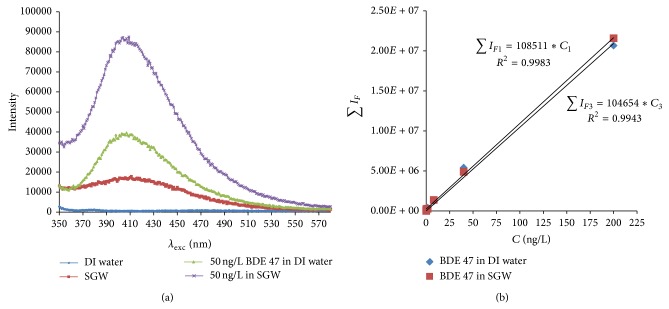
The effect of SGW on the fluorescence spectra of BDE 47 (a) and linear correlations between fluorescence intensity and BDE 47 concentrations (b).

Fluorescencemeasurement of PBDEs in groundwater may also be affected by other chemical constituents such as metal cations and inorganic anions [23]. To investigate such potential interference, fluorescence spectral measurements of a series of solutions with different BDE 47 concentrations in SGW were performed.  Figure 6 shows that fluorescence intensity of BDE 47 was only slightly enhanced (3.7%) in SGW compared to that in DI water, due to some unidentified interactions between BDE 47 and other chemical species present in the groundwater. Apparently, the effect of groundwater composition on BDE 47 determination was minor giving the close calibration curves in DI water and SGW after the background fluorescence signal from the SGW was removed.

### 3.7. Comparison with Conventional Method

GC methods are commonly used in measuring PBDEs in aqueous solutions. Prior to GC analysis, the target PBDEs compounds must be first extracted from water samples using continuous liquid/liquid extraction (CLLE), solid-phase extraction (SPE), or separation funnel extraction (SFE) [41]. Such extraction processes not only take time (20–60 min), but also create recovery problems that often lead to underestimation of the target compounds [42, 43]. In addition, after extraction, the low concentration PBDEs compounds have to be further concentrated and then used for PBDEs measurement by GC-ECD or GC-MS [41]. The fluorescence method described here had the similar LOD as GC or GC-EI-MS (low to a few ng/L) but showed great advantages with rapid (about 1 min) and direct measurement that avoids lengthy extraction steps and guarantees 100% recovery. In addition, the new method only requires a small amount (2–4 mL) of sample (Table 2) and eliminated the use of any other organic solvents as well as separation and concentration procedures.

It should be pointed out that although individual congeners of PBDEs show characteristic fluorescence peaks at different excitation and emission wavelengths, when more than one congener of PBDEs are present, the corresponding emission spectra will overlap (Table 1). This may be a challenge to unambiguously identify individual PBDEs species in aqueous samples containing mixed PBDEs congeners. Thus, identification and quantification of individual PBDEs congeners will require spectral deconvolution or prior knowledge of the types of PBDEs present. Secondly, the method has to correct the effect of environmental variables such as pH and HA and potential other fluorescence-emission chemicals with similar excitation and emission peaks. Nevertheless, the fast analysis and 100% recovery of the new method can be ideal for mechanistic study with known background solution chemical composition and under controlled environmental conditions. Under such conditions, quantitative spectral analyses are possible as the concentrations of interfering fluorescence species are well-defined. In addition, the fluorescence-based approach opens a way to design in situ observation systems and on-line detection systems to rigorously study PBDEs reactive transport in flow through systems such as in columns and flow cells [22, 26, 44].

## 4. Conclusions and Implication

This study developed a new method for rapid and direct measurements of PBDEs in aqueous samples using fluorescence spectroscopy. Results showed that tri- to deca-BDE (BDE 28, BDE 47, BDE 99, BDE 153, BDE 190, and BDE 209) that are commonly present in surface and groundwaters have distinct fluorescence spectra and peak positions that can be exploited to identify these species and determine their concentrations in aqueous solutions. Compared with conventional GC-based analytical methods, the fluorescence spectroscopy method is more efficient as it only uses a small amount of samples (4 mL), avoids lengthy concentration and extraction steps, and has a low detection limit: 1.71–5.82 ng/L for BDE 28, BDE 47, BDE 190, and BDE 209 and 45.55–69.95 ng/L for BDE 99 and BDE 153. The environmental variables including pH and HA contents can affect fluorescence intensity, but their effect can be corrected through proper spectral Deconvolution procedures if the interfering factors can be independently quantified. This method is particularly suitable for mechanistic studies of processes such as sorption and desorption and reactive transport of specific PBDEs species under controlled conditions. The method also has a potential for in situ investigation of reactive transport of PBDEs in porous media.

## Figures and Tables

**Table 1:** The excitation and emission peak positions (*λ*_max⁡_, nm) of six single congeners of PBDEs at variable emission wavelengths (*λ*_em_ = 380, 405, and 440 nm) and excitation wavelengths (*λ*_exc_ = 289, 302, 320, and 340 nm).

PBDEs	Excitation spectra			Emission spectra			
	(*λ*_max⁡_, 380 nm)	(*λ*_max⁡_, 405 nm)	(*λ*_max⁡_, 440 nm)	(*λ*_max⁡_, 289 nm)	(*λ*_max⁡_, 302 nm)	(*λ*_max⁡_, 320 nm)	(*λ*_max⁡_, 340 nm)
BDE 28	300, 380	302, 405	302, 440	403, 289	409, 302	412, 320	436, 340
BDE 47	300, 380	300, 405	302, 440	408, 289	407, 302	406, 320	434, 340
BDE 99	289, 380	289, 405	289, 440	412, 289	413, 302	412, 320	423, 340
BDE 153	289, 380	289, 405	289, 440	419, 289	420, 302	427, 320	429, 340
BDE 190	302, 380	314, 405	323, 440	425, 289	418, 302	422, 320	445, 340
BDE 209	302, 380	305, 405	337, 440	412, 289	408, 302	429, 320	445, 340

**Table 2:** Comparison of fluorescence and GC or GC-EI-MS for determining PBDEs in aqueous solutions.

Methods	Congener	Linearity range (ng/L)	Sample and volume	Extraction method^*^	Recovery (%)	LOQ^a^/LOD^b^ (ng/L)	Mesurement time (min)
GC-EI-MS	Tri- to hexa-BDE [43]	20–600	100 mL surface water	SBSE	99–106	1–32^a^ 0.4–9.6^b^	56.69
	BDE 47, 99, 100, and 153 [42]	4–150	10 mL ultrapure water spiked with 10 ng/L PBDEs	CPE	99–106	1-2^b^	15.50

GC-ECD	BDE 28, 47, 85, 99, 100, 153, and 154 [45]	0.1–100 for BDE 28, 47; 0.5–500 for others	5 mL Ultra Milli-Q water spiked with stock solution of PBDEs	SPE-DLLME	72–100	0.03–0.15^b^	36.67

UV-fluorescence	BDE 28, 47, 190, and 209^*≠*^	0.32–1000 for BDE 28; 0–1000 for BDE 47; 0.2–50 for BDE 190; 0.04–100 for BDE 209;	4 mL DI water spiked with stock solution of PBDEs	No need	100	1.71–5.82^b^	1
	BDE 99 and 153^*≠*^	0.064–2000				45.55–69.95^b^	1

^*^SBSE, stir bar sorptive extraction; CPE, cloud point extraction; HS-SPME, headspace solid-phase microextraction; SPE-DLLME, solid-phase extraction-dispersive liquid-liquid microextraction; GC-ITD-MS/MS, gas chromatography-ion trap tandem mass spectrometry.

^≠^In this study calculated by *I*_F(406 nm)_ = *a*_1_∗*C* + *b*_1_.

^a^The limit of quantification (LOQ).^b^The limit of detection (LOD).
